# Versatile and scalable fabrication method for laser-generated focused ultrasound transducers

**DOI:** 10.1364/OL.44.006005

**Published:** 2019-12-09

**Authors:** E. Aytac-Kipergil, E. J. Alles, H. C. Pauw, J. Karia, S. Noimark, A. E. Desjardins

**Affiliations:** 1 Department of Medical Physics and Biomedical Engineering, University College London, Malet Place Engineering Building, London WC1E 6BT, UK; 2 Wellcome/EPSRC Centre for Interventional and Surgical Sciences, Charles Bell House, University College London, 43-45 Foley Street, London W1W 7TY, UK

## Abstract

A versatile and scalable fabrication method for laser-generated focused ultrasound transducers is proposed. The method is based on stamping a coated negative mold onto polydimethylsiloxane, and it can be adapted to include different optical absorbers that are directly transferred or synthesized *in situ*. Transducers with a range of sizes down to 3 mm in diameter are presented, incorporating two carbonaceous (multiwalled carbon nanoparticles and candle soot nanoparticles) and one plasmonic (gold nanoparticles) optically absorbing component. The fabricated transducers operate at central frequencies in the vicinity of 10 MHz with bandwidths in the range of 15–20 MHz. A transducer with a diameter of 5 mm was found to generate a positive peak pressure greater than 35 MPa in the focal zone with a tight focal spot of 150 μm in lateral width. Ultrasound cavitation on the tip of an optical fiber was demonstrated in water for a transducer with a diameter as small as 3 mm.

Laser-generated focused ultrasound (LGFU) transducers utilize optically absorbing materials to produce ultrasound via the photoacoustic effect. High ultrasonic frequencies (>10MHz) and wide bandwidths (>7MHz) are obtained from photoacoustic generation [1]. By means of geometrical focusing, small focal spot sizes (<75μm) and pressures relevant to therapeutic applications can be achieved [2]. Thus, LGFU transducers are attractive for both high-precision ultrasound therapy and imaging [2–6].

Remarkable progress regarding generating LGFU transducers has been made recently. However, a key challenge with interventional applications remains in those areas where lateral diameters are highly constrained (e.g., <3mm): developing a fabrication method for transducers at this scale that can accommodate various optical absorbers for photoacoustic generation. To optimize the efficiency of transducing pulsed light to ultrasound, materials that exhibit high optical absorption, fast thermal diffusion, and large thermoelastic expansion capacities are required [2]. Several materials have previously been highlighted, including composites of optical absorbers and polymeric hosts. As an optically absorbing component, nanoparticles such as carbon black [7–9], carbon nanotubes (CNTs) [2,10–13], carbon nanofibers [14], candle soot nanoparticles (CSNPs) [15,16], reduced graphene oxide [17], and gold nanoparticles (AuNPs) [8,12,18,19] have been considered. The nano-scale dimensions of these absorbers enable rapid heat diffusion to the host medium. Host materials with high thermal expansion coefficients are often chosen to achieve high photoacoustic conversion efficiencies. As a host material, polydimethylsiloxane (PDMS) has been highlighted due to its high thermal expansion coefficient [20] ($\beta \approx 300\times 10^{-6}\;C^{-1}$), optical transparency (400–1100 nm) [21], and an acoustic impedance well matched to biological tissue [22].

There are several essential specifications in fabricating LGFU transducers. The substrate should be optically transparent and smooth relative to acoustic wavelengths [23]. In addition, the substrate must allow for uniform and robust adhesion to the optically absorbing material. Ideally, an LGFU fabrication method would also allow for a wide range of optical absorbers. Meeting these requirements has proven challenging [15,23,24]. To address these technical challenges, we present a scalable method for fabricating LGFU transducers based on stamping a coated negative mold onto PDMS. We demonstrate the versatility of the method by presenting LGFU transducers with outer diameters as small as 3 mm, incorporating various materials as optically absorbing layers created by both transfer and *in situ* synthesis steps. To transfer the optically absorbing material from the mold to the host, dip-coating, flame treatment, and membrane wrapping methods were considered. Carbonaceous [multiwalled carbon nanotubes (MWCNTs) and CSNPs] and plasmonic (AuNPs) nanoparticles were used separately as optical absorbers. Carbonaceous nanoparticles have broadband optical absorption. Plasmonic nanoparticles can have selective optical absorption so that they are substantially transmissive at other wavelengths for multimodal applications.

For achieving concave geometries, ball bearings with high degrees of sphericity and smoothness were chosen as negative molds (Grade 100 chrome steel; AISI 52100, Simply Bearings). High sphericity (variation in radius/diameter: ≤0.0025mm) leads to an ultrasound-generating surface of a desired geometry, and low surface roughness (≤0.127μm) ensures efficient absorber infiltration into the host. PDMS (Sylgard 184, Dowsil) was chosen as a host material and prepared following the manufacturer’s specifications. The mixture was left to degas in a vacuum chamber for ≈30min. Polytetrafluoroethylene (PTFE) tubes of varying diameters were fixed on a straight backing and filled with PDMS. As shown in Fig. 1(a), to create an optically absorbing layer, the mold was coated in various ways: i.e., (i) a “dip-coating method,” in which a ball bearing is dipped into functionalized MWCNTs dispersed in xylene [12] and left exposed under ambient conditions to remove the solvent; (ii) a “flame treatment method,” in which a ball bearing is placed within the flame core of a paraffin candle at ≈1cm above the wick for 4 to 10 s, depending on the diameter of the ball bearing; and (iii) a “membrane-wrapping method,” in which a ball bearing is encapsulated with a previously prepared PDMS composite film, i.e., PDMS-AuNPs [12,19] or PDMS-MWCNTs [25]. The coated ball bearing was then stamped onto the tubing filled with PDMS, and the assembly was cured at 100°C for 45 min [Fig. 1(a)]. In addition to the transfer-based methods explained above, an optically absorbing region was also created via *in situ* synthesis. The same fabrication steps were followed using an uncoated ball bearing, and the resulting PDMS structure with a concave surface was put into a gold salt solution [0.5% HAuCl4·3H2O (Sigma-Aldrich) in ethanol]. AuNPs were synthesized on the surface of the transducer [Fig. 1(b)]. The fabrication yields were 40%, 85%, and 80% for transducers with CSNPs, MWCNTs, and AuNPs, respectively. The optical absorption spectra of the LGFU transducers were characterized using an integrating sphere (FOIS-1, Ocean Optics, USA) and distal-end illumination from a broadband white light source (HL-2000-HP-FHSA, Ocean Optics, USA). The output light from the integrating sphere was spectrally resolved over a wavelength range of 450–1000 nm (Maya2000 Pro, Ocean Optics, USA) [Fig. 2(b)]. Both of the carbonaceous materials were found to have a broadband optical absorption >92% within the measured spectral range. The optical absorption of the AuNPs was wavelength selective [Fig. 2(b)]: for wavelengths below 600 nm, the AuNPs exhibited a high optical absorption >90% at 532 nm. From 850 nm upwards, the optical absorption decreased; it was <55% at 1000 nm. The transmission at longer wavelengths (>850nm) can be further enhanced by optimizing the synthesis time [12].

**Fig. 1.**
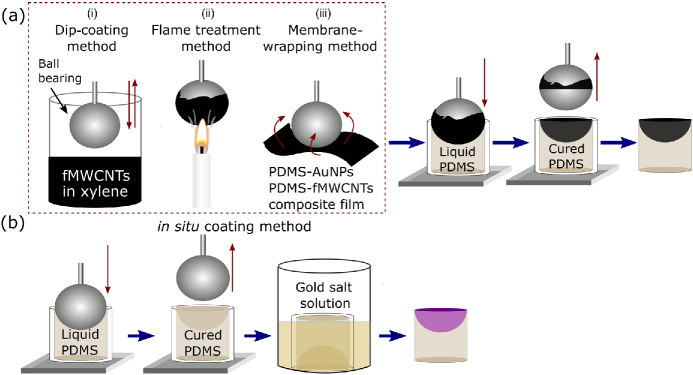
Fabrication process shown schematically, with an optically absorbing layer created with (a) transfer and (b) *in situ* synthesis.

**Fig. 2.**
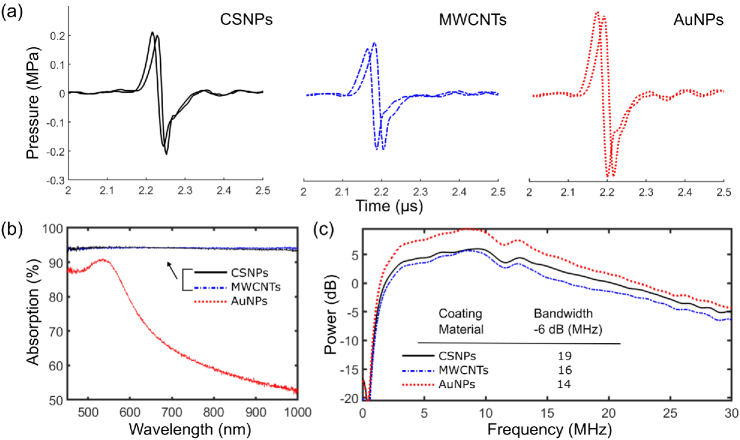
(a) Ultrasound pressure time series generated by two transducers of each type at their foci [two transducers with CSNPs (left), MWCNTs (middle), and AuNPs (right)]. (b) Optical absorption spectra of the materials (c) Power spectra of the A-scans.

To characterize the ultrasound generation of transducers with different materials, a pulsed laser (FQ-200-20-V-532, Elforlight, UK; 532 nm, 10 ns, 100 Hz, 40 μJ) was used. A needle hydrophone (200 μm; Precision Acoustics, UK) calibrated within the range from 1 to 30 MHz was used to measure generated ultrasound pressure amplitudes and bandwidths at the focal points of the transducers. Transducers with CSNPs, MWCNTs, and AuNPs were fabricated with the flame treatment method, the transfer method following dip coating, and the membrane-wrapping method, respectively. In terms of their pressure amplitudes, transducers with CSNPs and MWCNTs performed similarly (peak-to-peak pressure: 0.42, 0.37 MPa, respectively); a slightly higher amplitude was observed for transducers comprising AuNPs (peak-to-peak pressure: 0.56 MPa) [Fig. 2(a)]. CSNPs are more straightforward to fabricate but are subject to considerable variability in particle size distribution and coating thickness. The corresponding −6dB bandwidths for the transducers with CSNPs, MWCNTs, and AuNPs were 19, 16, and 14 MHz, respectively, with central frequencies of approximately 10 MHz [Fig. 2(c)]. Despite the differences in the optical absorbers used, the ultrasonic characteristics of different transducers were largely similar; differences may be ascribed in part to the inherent variability of fabrication steps. A different frequency range can be obtained by changing the thickness of the composite film [7,26]. However, if the film thickness is reduced, and the optical absorption coefficient is unchanged, less light will be absorbed, and therefore, the generated ultrasound pressure will be reduced. Alternatively, the ultrasound bandwidth can be increased by using an excitation light pulse with a shorter duration for a given transducer [27]. An additional consideration here is that acoustic attenuation in PDMS increases with frequency [7]. The frequency of the transducer can also be controlled indirectly through changing the absorption coefficient of the absorber. If the optical absorption coefficient is increased, the light will be absorbed over a smaller region, and the characteristic length will be decreased resulting in the generation of a larger bandwidth [28]. However, the temperature increase will be larger in a smaller volume, and at high pulse energies, the probability of damaging the coating will increase.

To demonstrate the scalability of our fabrication method, we used various geometrical specifications for transducer structure. This flexibility provides the advantage of meeting the varying requirements of diverse biomedical applications, such as the lateral dimensions available for the device, the spatial confinement of the focal spot, and the pressure amplitude at the focus. To estimate the thicknesses of the optical absorbing layers and to evaluate their homogeneity, cross sections of the transducers were imaged with optical microscopy (Fig. 3). The measurements showed that the coatings were highly uniform in thicknesses, with ±1μm variations along the surface. Successful integration of the absorbers into the host PDMS was observed for all of the tested transducers. Ultrasound bandwidths of the transducers with AuNPs were lower than those of carbon-based transducers overall [example shown in Fig. 2(c)], which is likely due to their greater coating thicknesses [7]. No gaps or delamination were observed between the membrane and substrate PDMS across the entire cross section of the transducers fabricated by the membrane-wrapping method. The absence of gaps is consistent with conformal wrapping of the films across the hemispherical surface.

**Fig. 3.**
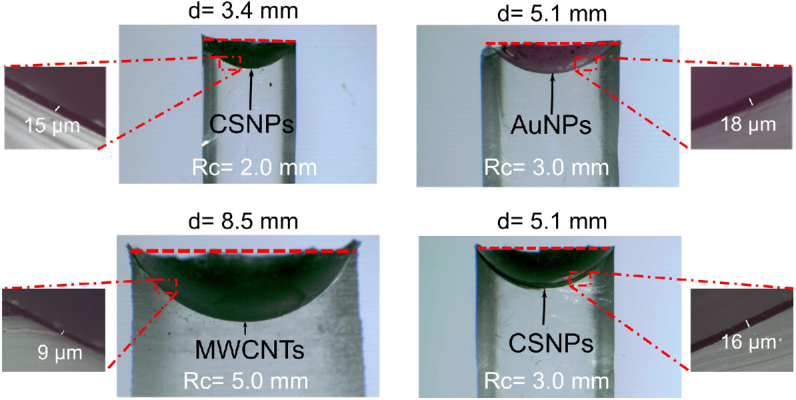
Optical microscopy of transducer cross sections [radius of curvature (Rc), diameter (d)].

To characterize the geometrical focusing of the transducers, acoustical field scans were performed. Fig. 4 shows the field scan for a transducer with AuNPs with a radius of curvature of 3 mm and a diameter of 5 mm and using the needle hydrophone across a 2.5mm×2.5mm grid at a 50 μm step size applying two orthogonal motorized stages (MTS50/M-Z8, Thorlabs, Germany). This hydrophone was positioned at an axial distance corresponding to the focal length; its signal was pre-amplified by 20 dB (DHPVA-200, Femto, Germany) and digitized (14 bits, 125 MS/s, M4i.4420-x8, Spectrum, Germany). The measured pressure field was subsequently numerically propagated to different axial distances using the angular spectrum approach [29] to determine the full-width-at-half maximum beam width at each parallel plane spaced at 25 μm [Fig. 4(b)]. The propagation showed that the narrowest beam waist of 150 μm was obtained at the geometrical focus of the transducer and that the beam exhibits circular symmetry.

**Fig. 4.**
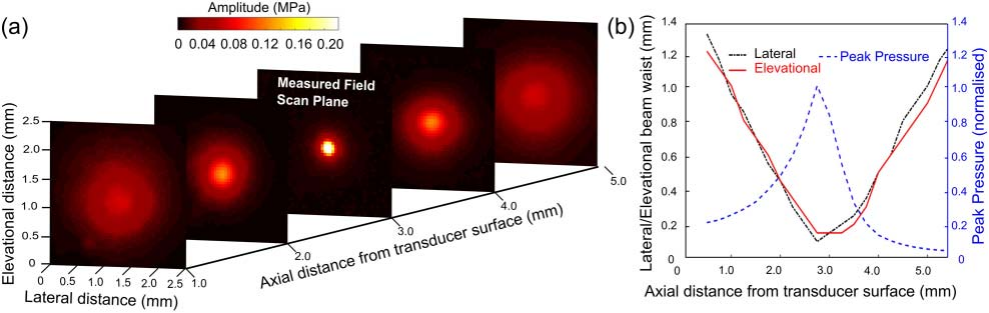
(a) Maximum intensity of the transmitted acoustic field measured at 3 mm axially, and the propagated fields (Rc=3mm; d=5mm). (b) Lateral and elevational extents of the acoustic beam and the normalized peak acoustic amplitude.

To characterize the performance of the fabricated LGFU transducers at high optical fluences, a pulsed Nd:YAG laser (1064 nm, Ultra 50, Quantel, France) with 8 ns pulse duration and 20 Hz repetition rate was used as an excitation source. To avoid damaging the needle hydrophone with high ultrasound pressures, a custom fiber-optic hydrophone (FOH) comprising a bare, flat-cleaved single-mode optical fiber was used to measure pressure amplitudes generated from high laser pulse energies (>4mJ). The FOH had an active sensing region determined by its core diameter (9 μm) and a sensitivity of approximately 7 mV/MPa that was determined at low ultrasound pressures by comparing signal amplitudes with those obtained using a calibrated reference hydrophone, as described previously [2]. The FOH was probed using a continuous wave laser (1500–1600 nm; TUNICS T100S-HP/CL, Yenista Optics, France). An optical circulator was used to deliver the light to the tip of the fiber and return the reflected light to a photodiode (DET01CFC, Thorlabs, Germany). The output of the photodiode was pre-amplified by 60 dB, digitized, and used to record the reflected time-varying optical power modulation produced by the incident ultrasound wave. A series of 128 A-scans was acquired without averaging to determine the pressure amplitudes and cavitation probabilities for different transducer geometries at various optical energies [Fig. 5(a)]. With 5-mm-diameter transducers, a pulse energy (PE) of 9.6 mJ yielded a photodiode output of over 260 mV [Fig. 5(c)], corresponding to the positive peak pressure. Using the detector with a sensitivity of 7 mV/MPa, these voltages correspond to pressure amplitudes greater than 35 MPa. Likewise, the 3-mm-diameter transducers produced positive peak pressures at the foci greater than 25 MPa.

**Fig. 5.**
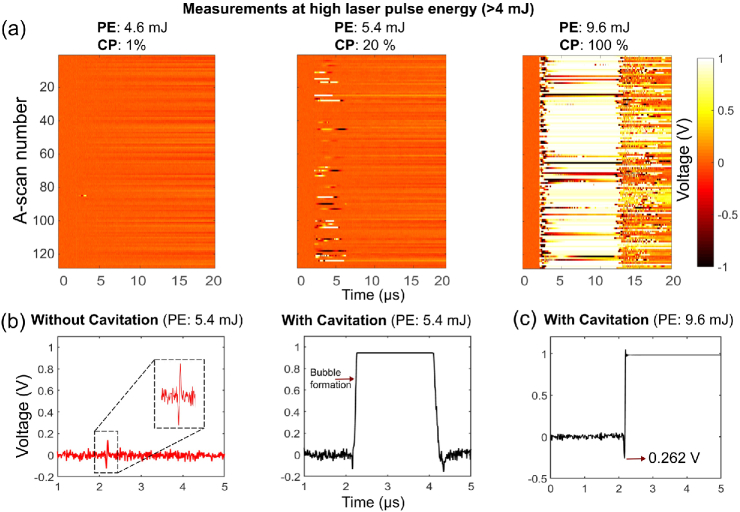
(a) Concatenated A-scans displayed as images. [Transducer (CSNPs): Rc=3mm and d=5mm; cavitation probabilities (CP)]. (b) A-scans with cavitation and without cavitation (PE=5.4mJ). (c) Sample A-scan when the PE was 9.6 mJ.

For a given transducer, there was a dependency of the cavitation probability on the fluence. When displayed as the photodiode voltage, the A-scans recorded with the FOH [Fig. 5(b)] appear inverted compared to those recorded with the needle hydrophone [Fig. 2(a)], as an increase in pressure resulted in a decrease in the refractive index mismatch between the FOH and water. When the negative peak pressure arrived, microbubble(s) were formed as a result of the rarefaction. The interrogation light within the FOH reflected from a glass–gas interface rather than a glass–liquid interface, resulting in a larger refractive index mismatch and consequently a larger positive signal that saturated the photodiode. By counting the number of A-scans exhibiting saturation, cavitation probabilities were calculated (Table 1). Cavitation at the focal zone was observed for all of the transducers with CSNPs, MWCNTs, and AuNPs. A cavitation probability of 100% was achieved for different geometries and optical absorbers. The maximum fluence ($54\;\mathrm{mJ}/{\mathrm{cm}}^{2}$) was below the damage thresholds of comparable composite materials reported elsewhere [2,8], and no optical damage or performance reduction was observed.

**Table 1.** Cavitation Probabilities (%) for Two CSNP-Based Transducers with Different Geometries

Fluence ($\mathrm{mJ}/{\mathrm{cm}}^{2}$)	Rc=3 and d=5mm	Rc=2 and d=3mm
23	1	0
27	20	0
31	98	0
39	100	11
46	100	76
54	100	100

In this Letter, we presented a new method for fabricating laser-generated ultrasound transducers with different geometries and optical absorbers using a stamping method. Optical microscopy measurements, feature-free field scans, nearly symmetric bi-polar temporal ultrasound profiles at the focus with corresponding broad bandwidths, and pressure levels qualitatively comparable to previous work confirmed that well-structured composites were formed within the transducers. Ultrasound cavitation was observed for transducers with diameters as small as 3 mm. To the best of our knowledge, they were the smallest LGFU transducers from which cavitation has been observed; crucially, their diameters allow for integration into a wide range of interventional devices. Key advantages of the fabrication method presented here are that the nanocomposite optically absorbing coating can have a controlled thickness and that it is in direct contact with the surrounding medium. In a previous study where a different method was used [15], an overlying PDMS layer may have contributed to significant ultrasound attenuation that led to sub-cavitation pressures and lower frequencies (−6dB range: 1.75–8.85 MHz). Another advantage is the ability to incorporate different optically absorbing materials, including dichroic coatings that will pave the way to simultaneous all-optical ultrasound and photoacoustic imaging, as well as simultaneous therapy and ultrasonic monitoring [12].
